# Value of CT-Textural Features and Volume-Based PET Parameters in Comparison to Serologic Markers for Response Prediction in Patients with Diffuse Large B-Cell Lymphoma Undergoing CD19-CAR-T Cell Therapy

**DOI:** 10.3390/jcm11061522

**Published:** 2022-03-10

**Authors:** Christian Philipp Reinert, Regine Mariette Perl, Christoph Faul, Claudia Lengerke, Konstantin Nikolaou, Helmut Dittmann, Wolfgang A. Bethge, Marius Horger

**Affiliations:** 1 Department of Radiology, Diagnostic and Interventional Radiology, University Hospital Tuebingen, Hoppe-Seyler-Str. 3, 72076 Tuebingen, Germany; regine.perl@med.uni-tuebingen.de (R.M.P.); konstantin.nikolaou@med.uni-tuebingen.de (K.N.); marius.horger@med.uni-tuebingen.de (M.H.); 2 Department of Hematology, Oncology, Clinical Immunology and Rheumatology, University Hospital Tuebingen, Hoppe-Seyler-Str. 3, 72076 Tuebingen, Germany; christoph.faul@med.uni-tuebingen.de (C.F.); claudia.lengerke@med.uni-tuebingen.de (C.L.); wolfgang.bethge@med.uni-tuebingen.de (W.A.B.); 3 Cluster of Excellence iFIT (EXC 2180) Image Guided and Functionally Instructed Tumor Therapies, University of Tuebingen, 72074 Tuebingen, Germany; 4 Department of Radiology, Nuclear Medicine, University Hospital Tuebingen, Hoppe-Seyler-Str. 3, 72076 Tuebingen, Germany; helmut.dittmann@med.uni-tuebingen.de

**Keywords:** diffuse-large B cell lymphoma, chimeric antigen receptor T cells, response assessment, positron emission tomography, computed tomography, texture analysis

## Abstract

The goal of this study was to investigate the value of CT-textural features and volume-based PET parameters in comparison to serologic markers for response prediction in patients with diffuse large B-cell lymphoma (DLBCL) undergoing cluster of differentiation (CD19)-chimeric antigen receptor (CAR)-T cell therapy. We retrospectively analyzed the whole-body (WB)-metabolic tumor volume (MTV), the WB-total lesion glycolysis (TLG) and first order textural features derived from ^18^F-FDG-PET/CT, as well as serologic parameters (C-reactive protein [CRP] and lactate dehydrogenase [LDH], leucocytes) prior and after CAR-T cell therapy in 21 patients with DLBCL (57.7 ± 14.7 year; 7 female). Interleukin 6 (IL-6) and IL-2 receptor peaks were monitored after treatment onset and compared with patient outcome judged by follow-up ^18^F-FDG-PET/CT. In 12/21 patients (57%), complete remission (CR) was observed, whereas 9/21 patients (43%) showed partial remission (PR). At baseline, WB-MTV and WB-TLG were lower in patients achieving CR (35 ± 38 mL and 319 ± 362) compared to patients achieving PR (88 ± 110 mL and 1487 ± 2254; *p* < 0.05). The “entropy” proved lower (1.81 ± 0.09) and “uniformity” higher (0.33 ± 0.02) in patients with CR compared to PR (2.08 ± 0.22 and 0.28 ± 0.47; *p* < 0.05). Patients achieving CR had lower levels of CRP, LDH and leucocytes at baseline compared to patients achieving PR (*p* < 0.05). In the entire cohort, WB-MTV and WB-TLG decreased after therapy onset (*p* < 0.01) becoming not measurable in the CR-group. Leucocytes and CRP significantly dropped after therapy (*p* < 0.01). The IL-6 and IL-2R peaks after therapy were lower in patients with CR compared to PR (*p* > 0.05). In conclusion, volume-based PET parameters derived from PET/CT and CT-textural features have the potential to predict therapy response in patients with DLBCL undergoing CAR-T cell therapy.

## 1. Introduction

Diffuse large B-cell lymphoma (DLBCL) is the most common subtype of non-Hodgkin’s lymphoma in adults with a prevalence of almost 40% [1]. Standard treatment regimens are efficacious, but up to 15% of patients will exhibit primary refractory disease and another 30–35% will experience relapse after initial response [2,3]. In the refractory/relapsed DLBCL patients, incidence of disease recurrence is high, even after salvage therapy combined with autologous stem cell support, leading to a dismal long-term survival rate [4]. In this setting, novel treatment strategies are explored. A promising, emerging therapy option are CD19 CAR (chimeric antigen receptor)-T cells, which consist of genetically modified autologous T cells by retroviral or lentiviral vectors containing DNA encoding a CAR [1]. It has been shown that CD19 CAR-T cells provide high and durable response rates even in refractory and relapsed DLBCL [5,6,7].

Imaging is playing a major role for diagnosis and treatment monitoring in patients with DLBCL, and the most frequently recommended technique is the positron emission tomography (PET) using ^18^F- Fluorodeoxyglucose (FDG) as a tracer targeting glucose metabolism mostly in combination with computed tomography (CT) [8]. Hence, morphologic and glucometabolic changes in tumor herald either lymphoma response or relapse. The latter has been further refined by calculating the entire metabolic tumor volume (MTV) and the total lesion glycolysis (TLG). Both PET and CT image data can be additionally analyzed by using texture analysis with potential for tumor characterization either in the primary setting (baseline) for prognosis evaluation, or during therapy as an adjunct to morphologic and metabolic changes for more accurate response assessment [9,10]. Finally, some laboratory biomarkers like lactate dehydrogenase (LDH) in serum have been used for a long time as prognostic factors in lymphoma patients.

CD19-CAR T cell therapy as immunotherapy leads to a strong immunological response with T cell activation and cytokine release as illustrated by the most commonly observed adverse effect: cytokine release syndrome (CRS) and immune effector cell-associated neurotoxicity syndrome (ICANS). Manifestation of CRS goes along with hypersecretion of IL6, IL2-R, ferritin and CRP from activated macrophages following CAR-T cell activation [11].

Hence, the intention of this study was to investigate the value of CT-textural features and volume-based PET parameters in comparison to serologic markers for response prediction in patients with DLBCL undergoing CD19-CAR-T cell therapy.

## 2. Material and Methods

This retrospective image and laboratory data analysis was approved by the local ethics committee and the patients waived written consent (project number 277/2020BO2).

### 2.1. Patient Characteristics

Twenty-one consecutive patients with DLBCL (mean age 57.7 ± 14.7 year; seven female) undergoing CAR-T cell therapy from 06/2018 to 02/2021 were retrospectively evaluated. All patients had pathologically confirmed DLBCL. According to Ann-Arbor classification, 2/21 patients had a stage I, 3/21 patients had stage II, 5/21 patients had stage III and 11/21 patients were classified stage IV. According to the International Prognostic Index (IPI) scoring system, 3/21 patients were rated score 1, 9/21 patients were rated score 3 and 9/21 patients were rated score 4. Patient characteristics are summarized in Table 1.

At baseline staging, before CAR-T cell therapy, 17/21 patients underwent ^18^F-FDG-PET/CT and 4/21 patients underwent CT using standardized protocols. All patients were additionally monitored by serologic parameters. At first follow-up after CAR-T cell therapy, 18/21 patients underwent ^18^F-FDG-PET/CT and 3/21 patients underwent CT.

Therapy response was classified according to the Lugano classification system including CT-based response criteria and the Deauville five-point scale (Deauville 5PS) scoring system [12]. Each FDG-avid (or previously FDG-avid) lesion was rated independently by two readers. Response to CAR-T cell therapy was defined as PR in case of metabolically active disease (Deauville score 4), which is defined as lymphoma manifestations with ^18^F-FDG-uptake slightly to moderately higher than the liver background ^18^F-FDG-uptake without metric progression on CT. In case of no measurable ^18^F-FDG-uptake and ^18^F-FDG-uptake below or equal to uptake in the liver (Deauville score 1–3), CR was assigned.

### 2.2. Imaging Protocols

All PET/CT examinations were performed on a state-of-the art clinical scanner (Biograph mCT^®^, Siemens Healthineers, Erlangen, Germany). All patients fasted overnight before examination. Approximately 300 MBq ^18^F-FDG were injected intravenously 60 min prior to image acquisition. Standardized CT examination protocols included weight-adapted 90–120 mL intravenous CT contrast agent (Ultravist 370^®^, Bayer Vital, Leverkusen, Germany). Portal-venous phase acquisitions were obtained with 70 s delay time using a tube voltage of 120 kV and a reference dose of 200 mAs. Image reconstruction was performed using iterative CT reconstruction (Siemens SAFIRE^®^, Forchheim, Germany). PET was acquired from the skull to the mid-thigh level over six to eight bed positions and reconstructed using a 3D ordered subset expectation maximization algorithm (two iterations, 21 subsets, Gaussian filter 2.0 mm, matrix size 400  ×  400, and slice thickness 2.0 mm). PET acquisition time was 2–3 min per bed position.

CT was performed with patients in the supine position using a 128-slice MDCT scanner (SOMATOM Definition Flash, Siemens Healthcare, Erlangen, Germany). Contrast-enhanced portal-venous phases were obtained using 120-kV photon energy, 200-mAs tube current, a soft tissue image reconstruction kernel, and 1-mm slice thickness for image reconstruction. A weight-adapted iodine contrast agent (Ultravist [Iopromide] 370, Bayer Vital, Leverkusen, Germany) was given intravenously at a rate of 2 mL/s followed by a 30-mL saline chaser. Image acquisition began 70 s after the start of contrast agent injection. Image reconstruction was performed in all patients using filtered back projection.

### 2.3. Quantitative Image Analysis

The segmentation of lymphoma manifestations was performed by one reader using approved software for quantification of PET parameters on Syngo.via VB 30A (Siemens Healthineers, Erlangen, Germany). Evaluation included all lesions which were characterized by increased ^18^F-FDG uptake above liver background activity (Deauville score 4). Segmentation of each lesion was performed manually using 50%-isocontour volumes of interests (VOIs) for quantification. Whole-body MTV and whole-body TLG were calculated as the sum of all quantified lymphoma manifestations per patient.

CT-texture analysis (CTTA) was performed in contrast-enhanced CT images derived from whole-body CT or whole-body ^18^F-FDG-PET/CT obtained in the portal-venous enhancement phase using a standardized protocol and dedicated radiomics software (Siemens Healthcare, Erlangen, Germany) that is based on the pyradiomics package, a python package for the extraction of radiomics features from medical imaging [13]. A slice thickness of 1 mm was used. Regions of interests (ROIs) were drawn manually in lymphatic tissue carefully excluding neighboring tissues like blood vessels. This standardized procedure of ROI setting was performed by a radiologist with five years of experience in CTTA. Standardized measurements were performed to provide comparability for all data sets. All set ROIs were used to generate specific VOIs. The first step consisted of image filtration for selectively extracting features of different sizes and intensity variation. In the 2nd step, quantification of tissue texture followed. The computation of each texture type for an input VOI involved assigning a new value (“texture value”) to all voxel of that VOI and thus creating a “texture image”. This involved the creation of a three-dimensional VOI within the largest lymph node, the features of which were used to calculate texture values on a fine spatial scale. Computation was performed on the current voxel and its neighborhood, and the results of that were stored as the current voxel’s texture value. To ensure reliable statistics for this patient cohort, we limited analysis of textural parameters to the following 1st order features which describe the distribution of voxel intensities within the mask through commonly used and basic metrics: mean, uniformity, entropy, skewness. The definitions of these textural parameters are provided in Table 2.

### 2.4. Laboratory Parameters

Laboratory parameters were extracted from the clinical data base on the same day as imaging. The upper limits of the reference ranges were: 250 U/L for serum LDH, 0.5 μg/dL for CRP, 130 U/L for AP, 4100–11,800 1/μL for leucocyte count, 158–613 U/mL for soluble IL-2R and 0–4 ng/L for IL-6.

### 2.5. Statistical Analysis

Statistical analysis was performed using SPSS Version 22 (IBM Corporation, Armonk, NY, USA). We tested all parameters for the normality with a Kolmogorov-Smirnov test. A Mann-Whitney-U test was used to test the differences in tumor volumetric parameters, 1st order textural features and laboratory parameters between the patient groups achieving CR or PR. To address the multiple comparisons, a Benjamin Hochberg correction was applied. The adjusted *p*-values were considered significant at a level of 0.05.

## 3. Results

In 12/21 DLBCL patients (57%), CR was observed after CAR-T cell therapy, whereas 9/21 patients (43%) showed PR. No patient had a progression at first follow-up after CAR-T cell therapy.

### 3.1. Quantitative Analysis of Tumor Volumetric Parameters

**Figure 1:**
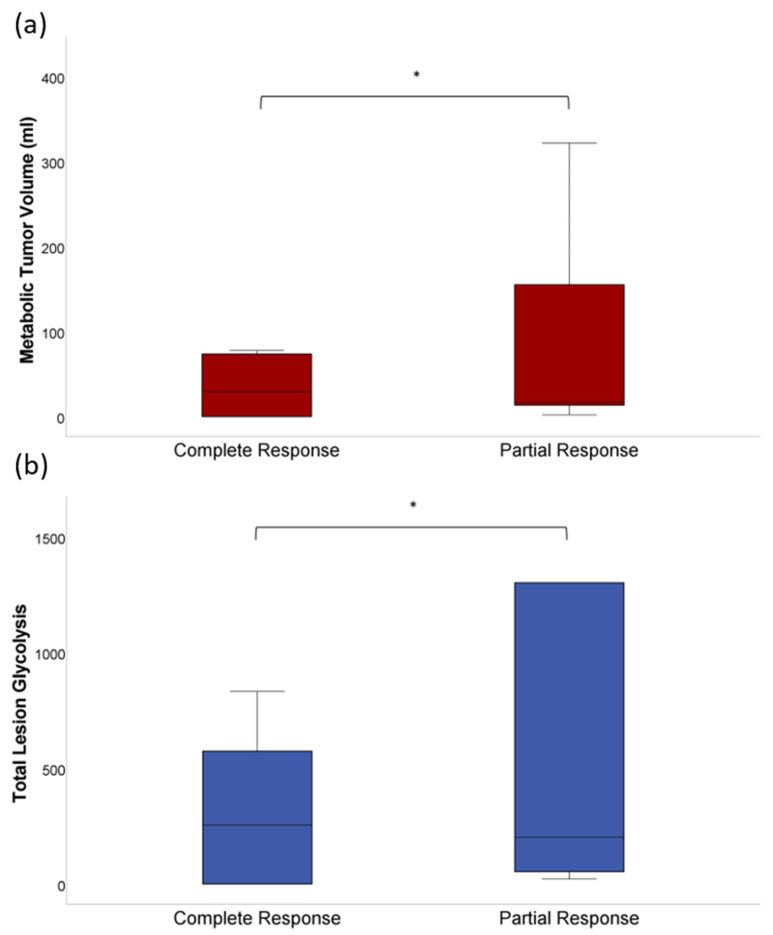
(**a**) At baseline ^18^F-FDG PET/CT before CAR-T cell therapy, both the whole-body MTV (35 ± 38 mL vs. 88 ± 110 mL) and (**b**) the whole-body TLG (319 ± 362 vs. 1488 ± 2254) were lower in patients achieving CR compared to patients achieving PR (*p* < 0.01 and *p* < 0.05). The asterisk (*) indicates clinical significance (*p* < 0.05).

The whole-body MTV and whole-body TLG differed significantly (*p* < 0.01 and *p* < 0.05, respectively) between the subgroups assigned to CR or PR showing lower levels in the CR group at baseline (MTV: 35 ± 38 mL vs. 88 ± 110 mL; TLG: 319 ± 362 vs. 1488 ± 2254) (Figure 1).

**Figure 2:**
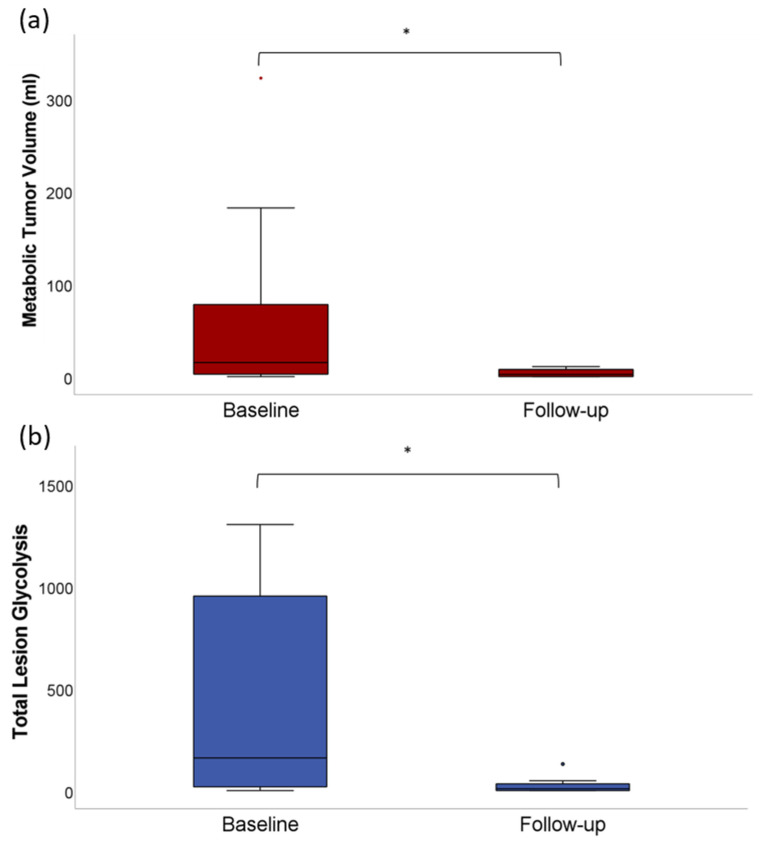
(**a**) Whole-body MTV at baseline ^18^F-FDG-PET/CT before CAR T cell therapy (62 ± 86 mL) and at first follow-up ^18^F-FDG-PET/CT (4 ± 5 mL) after CAR T cell therapy in the entire patient cohort (*p* < 0.01). (**b**) Whole-body TLG at baseline ^18^F-FDG-PET/CT before CAR-T cell therapy (925 ± 1722) and at first follow-up ^18^F-FDG-PET/CT (33 ± 59) after CAR-T cell therapy in the entire patient cohort (*p* < 0.01). The asterisk (*) indicates clinical significance (*p* < 0.05).

In the entire patient cohort, a significant (*p* < 0.01) decrease was observed for whole-body MTV (62 ± 86 mL vs. 4 ± 5 mL) and whole-body-TLG (925 ± 1722 vs. 33 ± 59) (Figure 2). After CAR-T cell therapy, MTV and TLG were not measurable in the subgroup achieving CR, whereas in the subgroup with PR, MTV (88 ± 110 mL vs. 7 ± 4 mL) and TLG (1488 ± 2254 vs. 41 ± 39) significantly decreased (*p* < 0.01).

### 3.2. CT-Texture Analysis

**Figure 3:**
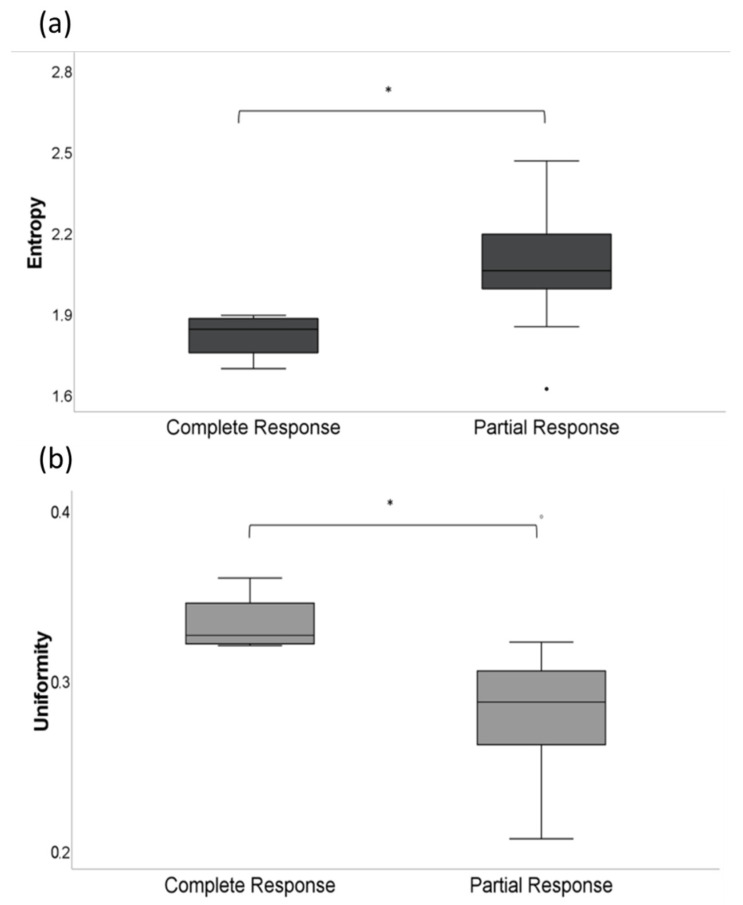
(**a**) Entropy at baseline imaging before CAR-T cell therapy in patients achieving CR (1.81 ± 0.1) vs. PR (2.08 ± 0.2; *p* < 0.05). (**b**) Uniformity at baseline imaging before CAR-T cell therapy in patients achieving CR (0.33 ± 0.02) vs. PR (0.28 ± 0.47; *p* < 0.05). The asterisk (*) indicates clinical significance (*p* < 0.05).

At baseline, the “entropy” proved lower (1.81 ± 0.1) in patients achieving CR compared to patients achieving PR (2.08 ± 0.2; *p* < 0.05) (Figure 3a). In contrast, the “uniformity” was higher in patients with CR (0.33 ± 0.02) compared to patients with PR (0.28 ± 0.47; *p* < 0.05) (Figure 3b). No significant differences were found for “skewness” (0.11 ± 0.21 [CR] vs. −0.32 ± 1.23 [PR]; *p* > 0.05) and “mean” (66.1 ± 18.89 [CR] vs. 49.3 ± 23.9 [PR]; *p* > 0.05) with a trend in “mean” for higher values in patients with CR.

### 3.3. Laboratory Parameters

**Figure 4:**
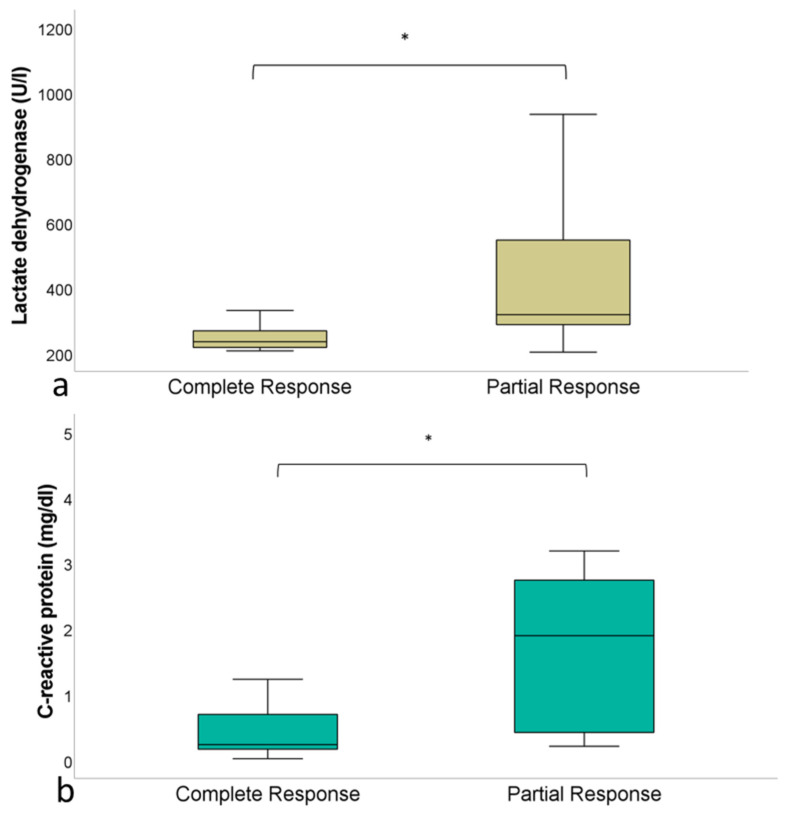
(**a**) Serum LDH at baseline before CAR-T cell therapy in patients achieving CR (240 ± 28 U/L) vs. patients achieving PR (443 ± 262 U/L; *p* < 0.05). (**b**) CRP at baseline before CAR-T cell therapy in patients achieving CR (2.6 ± 4.6 μg/dL) vs. patients achieving PR (4.2 ± 6.6 μg/dL; *p* < 0.05). The asterisk (*) indicates clinical significance (*p* < 0.05).

In the group of patients achieving CR after CAR-T cell therapy, the mean serum LDH proved significantly lower compared to the PR group at baseline (240 ± 28 U/L [CR] vs. 443 ± 262 U/L [PR]; *p* < 0.05) (Figure 4a). The CRP levels in serum at baseline also proved significantly lower in the CR group compared to the PR group (2.6 ± 4.6 μg/dL [CR] vs. 4.2 ± 6.6 μg/dL [PR]; *p* < 0.05) (Figure 4b). At baseline, no differences were found for the leucocyte count between patients achieving CR (5127 ± 766 1/μL) and PR (5290 ± 3142 1/μL; *p* > 0.05). 

In the whole patient cohort, we observed a significant decrease of CRP (2.8 ± 5.6 μg/dL vs. 0.1 ± 0.2 μg/dL; *p* < 0.01) and leucocyte count (5348 ± 2514 1/μL vs. 2954 ± 2024 1/μL; *p* < 0.01) and trend towards lower serum LDH (366 ± 235 U/L vs. 242 ± 69 U/L; *p* > 0.05) after CAR-T cell therapy.

**Figure 5:**
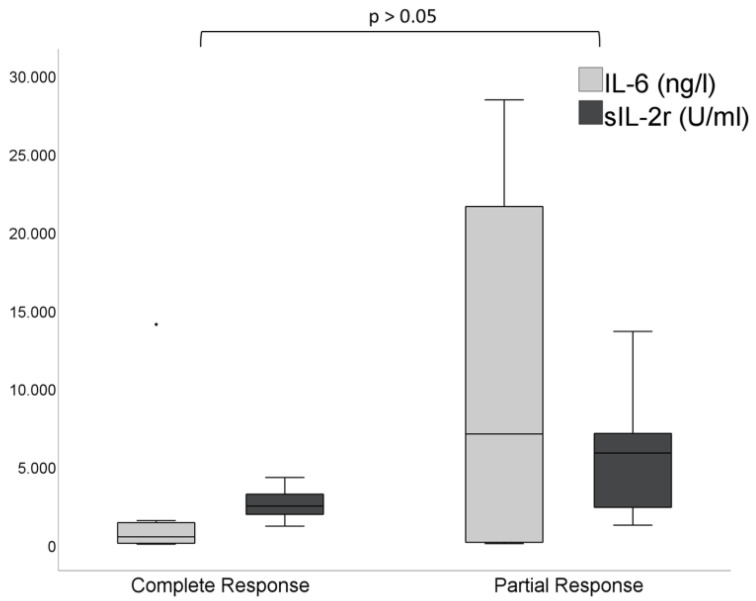
IL-6 peak (867 ± 951 ng/L vs. 9121 ± 11,266 ng/L) and IL-2R peak (2483 ± 1164 U/mL vs. 5548 ± 3949 U/mL) in patients with CR vs. PR (*p* > 0.05).

The mean time interval between re-infusion of CAR-T cells and IL-6 peak was 6.5 ± 4.3 days and between re-infusion of CAR-T cells and Il-2R peak the time was 7.4 ± 4.1 days. The IL-6 peak (867 ± 951 ng/L vs. 9121 ± 11,266 ng/L) and IL-2R peak (2483 ± 1164 U/mL vs. 5548 ± 3949 U/mL) were lower in patients with CR compared to PR, without statistical significance (*p* > 0.05) (Figure 5).

After CAR-T cell therapy, 20/21 patients developed a cytokine release syndrome (CRS). Of these, 10/21 patients had a CRS grade I, 7/21 patients had a CRS grade II, 2/21 patients had a CRS grade III and 1/21 patient had a CRS grade IV. In the group of patients with CR after CAR-T cell therapy, two patients had a manifest CRS (grade > 1), whereas eight patients with PR developed a manifest CRS.

## 4. Discussion

In this study, we investigated the predictive value of textural features derived from CT and volume-based parameters derived from ^18^F-FDG-PET/CT in comparison to serologic markers in patients with DLBCL undergoing CD19-CAR-T cell therapy.

Patients achieving PR had a considerably higher MTV and TLG in the baseline setting compared to patients achieving morphological and metabolic CR. This is not surprising, as the MTV was already found to have predictive value in lymphoma [14,15,16]. Xie et al. reported a negative progression-free survival (PFS) in patients with DLBCL presenting high MTV and SUV_max_ values in the pre-treatment setting, irrespective of the applied treatment regimen [14]. Similarly, Zhou et al. described MTV as the only independent predictor of progression-free survival and overall survival in their patient cohort with DLBCL undergoing R-CHOP therapy [15]. In a subsequent analysis, the same authors found baseline TLG to be a significant predictor of PFS [16]. Quantification of the volumetric parameters MTV and TLG and calculation of cut-off values for response prediction were also performed by Xie and Sasanelli [16,17]. Furthermore, response prediction based on MTV was performed in patients with follicular lymphoma undergoing immuno-chemotherapy [18].

Expectedly, quantitative ^18^F-FDG-PET proved also to be a good therapy monitoring tool with significant reduction in glucose metabolism in patients achieving PR and no residual uptake in patients achieving metabolic CR.

Other quantitative predicting and response monitoring metric features are those derived from texture analysis of either PET-metabolic or CT-morphologic image data, which are then post-processed by using radiomics parameters [13]. At this point, our results obtained from CT-radiomics analysis indicate that with increasing tumor tissue homogeneity and correspondingly decreasing heterogeneity in the baseline setting, the chances of achieving CR significantly increase. Similar results were reported in DLBCL patients undergoing immunochemotherapy [10]. Aide et al. demonstrated that ^18^F-FDG-PET heterogeneity of the largest lymphoma lesion was an independent predictor of two years-event-free survival [10]. In this study, the only independent predictor when analyzed together with IPI and MTV was the long-zone high-grade level emphasis.

Texture analysis was further demonstrated to have potential in improving the value of pretreatment PET/CT for prediction of the interim response of primary gastrointestinal-DLBCL [9]. In this report, the SUV_max_, MTV, as well as the entropy were significantly higher in the non-CR group. In the report by Aide et al., texture analysis of the skeleton in patients with DLBCL proved beneficial for diagnosis of infiltration with skewness being the only independent predictor of PFS [19].

The assumption that images contain information of disease-specific processes is the basis for the use of radiomics, which aims at enhancing the existing data by means of advanced mathematical analysis. Various studies from different fields in imaging highlight the potential of radiomics to support clinical decision-making [20,21]. However, various technical factors may influence the extracted radiomic features, which is a limitation of this approach that needs to be considered [22].

Although radiomics applications have yet to arrive in routine clinical practice, image interpretation using radiomics has potential in terms of a more personalized medicine in the future.

Another discipline in which radiomics is now increasingly being applied is pathology [23]. The idea to genetically classify tumors without biopsy using non-invasive extraction of image information promises support in diagnostics, individualized prognosis and therapy planning.

LDH is a non-specific marker for lymphoma whose prognostic significance is well established for both indolent and aggressive lymphomas at the time of diagnosis, which indirectly reflects the tumor burden. As expected, we found lower serum levels of LDH at baseline in DLBCL patients, which reached CR after CAR-T cell therapy. LDH significantly decreased after therapy accompanied also by decreasing serum levels of CRP and leucocyte count.

Of interest, the IL-6 and IL-2R serum peaks measured in the first week after CAR-T cell therapy onset proved lower in patients classified CR compared to patients classified PR. Almost half of our patient cohort developed a manifest CRS (grade 2 or higher) after CAR-T cell therapy, which was mainly observed in patients with PR after treatment.

Our results are in line with already existing data with respect to response prediction in DLBCL showing similar results of studies exploring the role of PET/CT and CT in immunotherapy.

Our study has some limitations. First, not all patients received the same CD19-CAR-T cells compound. Second, due to the retrospective study design, a selection bias or other confounding factors cannot be excluded. Third, the histologic subtype of DLBCL was not considered because of the small size of the entire cohort hampering an otherwise statistical analysis. Our observations have to be confirmed by larger prospective studies using multivariate analyses which include tumor volumetric and serologic markers.

In conclusion, volume-based PET parameters derived from PET/CT and CT-textural features have the potential to predict therapy response in patients with DLBCL undergoing CAR-T cell therapy.

## Figures and Tables

**Table 1:** Patient characteristics.

	I	II	III	IV
Ann-Arbor stage	2	3	5	11
International Prognostic Index Score	3	0	9	9
Cytokine Release Syndrome Grade	10	7	2	1

**Table 2:** Definitions of measured 1st order textural features *.

1st Order Feature	Definition
**Entropy**	Specifies the uncertainty/randomness in the image values. Measures the average amount of information required to encode the image values.
**Mean**	Mean of voxel intensities within the region
**Skewness**	Measures the asymmetry of the distribution of values about the mean value. Depends on where the tail is elongated and where the mass of the distribution is concentrated Can be positive or negative.
**Uniformity**	Sum of the squares of each intensity value. Measure of the homogeneity of the image array, where a greater uniformity implies a greater homogeneity or a smaller range of discrete intensity values.

* Describe the distribution of voxel intensities within the image region defined by the mask through commonly used and basic metrics.

## Data Availability

The data presented in this study are available on request from the corresponding author. The data are not publicly available due to privacy and ethical restrictions.

## Abbreviations

CAR	chimeric antigen receptor
CD19	cluster of differentiation 19
CR	complete remission
CRS	cytokine release syndrome
CTTA	CT texture analysis
Deauville 5PS	Deauville five-point scale
DLBCL	diffuse large B-cell lymphoma
FDG	^18^F- Fluorodeoxyglucose
ICANS	immune effector cell-associated neurotoxicity
IL	interleukin
IPI	international prognostic index
LDH	lactate dehydrogenase
MBq	megabecquerel
MTV	metabolic tumor volume
PFS	progression-free survival
PR	partial remission
R-CHOP	Rituximab in combination with cyclophosphamide, doxorubicin, vincristine, and prednisone
ROI	region of interest
SUV	uptake value
TLG	total lesion glycolysis
VOI	volume of interest
